# TSH promotes adiposity by inhibiting the browning of white fat

**DOI:** 10.1080/21623945.2020.1783101

**Published:** 2020-06-24

**Authors:** Jianmei Zhang, Huixiao Wu, Shizhan Ma, Ling Gao, Chunxiao Yu, Fei Jing, Jiajun Zhao

**Affiliations:** a Department of Endocrinology, Shandong Provincial Hospital Affiliated to Shandong First Medical University, Shandong, P.R. China; b Department of Endocrinology, Shandong Provincial Hospital Affiliated to Shandong University, Shandong, P.R. China; c Shandong Provincial Key Laboratory of Endocrinology and Lipid Metabolism, Shandong, P.R. China; d Department of Geriatrics, Weihai Municipal Hospital Affiliated to Shandong University; e Institute of Endocrinology and Metabolism, Shandong Academy of Clinical Medicine, Shandong, P.R. China

**Keywords:** Adiposity, browning, TSH, AMPK, PRDM16, PGC1Α

## Abstract

Adiposity is caused by an imbalance between energy intake and consumption. Promotion of the browning of white fat increases energy expenditure and could combat adiposity. Thyroid-stimulating hormone (TSH) has been confirmed to positively correlate with adiposity. However, the putative connection between TSH and white adipose browning has never been explored. In this study, we sought to assess the effect of TSH on white adipose tissue browning and energy metabolism. Subclinical hypothyroidism mice, thyroid-specific *Tshr*-knockout mice injected with TSH, adipocyte-specific and global *Tshr-*knockout micewere subjected to morphological, physiological, genetic or protein expression analyses and metabolic cages to determine the role of TSH on the browning of white adipose tissue and metabolism. 3T3-L1 and primary SVF cells were used to verify the effects and mechanism of TSH on the browning of white adipocytes. We show that increased circulation TSH level decreases energy expenditure, promotes adiposity, impairs glucose and lipid metabolism. Knockout of *Tshr* decreases adiposity, increases energy expenditureand markedly promotes the development of beige adipocytesin both epididymal and inguinal subcutaneous white fat via a mechanism that likely involves AMPK/PRDM16/PGC1α. Our results reveal an important role of TSH in regulating energy balance and adiposity by inhibiting the browning of white fat.

## 1. Introduction

The high prevalence of adiposity and its comorbidities have inspired an increase in research on adipose tissues. There are three types of adipose tissue. White adipose tissue (WAT) is characterized by large unilocular lipid-droplet-containing white adipocytes, which are generally believed to store excess energy and act as an active endocrine organ that secretes adipocytokines to regulate diverse physiological functions. Brown adipose tissue (BAT) is morphologically and functionally different from WAT. Brown adipocyte is composed of multiple lipid droplets and rich in mitochondria that express uncoupling protein 1 (UCP1). BAT specializes in energy consumption via thermogenesis to participate in maintaining body temperature [1]. This process depends on UCP1, which uncouples mitochondrial oxidative phosphorylation to produce heat [2]. Beige or brite adipocytes are distributed in classical WAT depots and share some characteristics with brown adipocytes. Beige adipocytes have low basal levels of UCP1 in their mitochondria and, when activated, can express UCP1 at high levels, similarly to classic brown adipocytes [3].

Browning, which is defined as stimulating the development of beige adipocytes in WAT, might ameliorate the adverse effects of excessive WAT and could help improve metabolic health [4,5]. The development of adiposity is associated with the balance between BAT and WAT; BAT dissipates energy as heat, while WAT stores excess energy. In some rodent models, a propensity towards adiposity is associated with decreased BAT activity, while resistance to adiposity correlates with increased BAT function or the development of beige cells into white adipocytes [6].

Thyroid-stimulating hormone (TSH) is a type of hypophyseal hormone. TSH receptors (*Tshrs*) have been identified in many tissues in addition to the thyroid, including adipose tissues, brain, kidneys, testes, heart, thymus and bone [7], indicating that TSH might exhibit other biological effects in addition to its functions on the thyroid gland. A large number of studies have demonstrated the correlation between serum TSH levels and adiposity. In some epidemiological studies, serum TSH levels are positively associated with body mass index (BMI) in euthyroid subjects [8]. Serum TSH levels and degree of obesity are positively correlated in patients with subclinical hypothyroidism or metabolic syndrome [9,10]. In addition, TSH levels are higher in obese patients than in non-obese patients [11].

However, the putative connection between TSH and white adipose browning has not been fully elucidated. In this study, we report that elevated TSH increases adiposity by reducing energy expenditure and that fat-specific or global *Tshr* knockout limits weight gain, resists high-fat diet-induced adiposity and promotes the browning of both epididymal white adipose tissue (eWAT) and inguinal subcutaneous white adipose tissue (iWAT), likely via AMPK/PRDM16/PGC1α. We identify an important role for TSH in adiposity and energy metabolism through inhibition of the browning of white fat.

## 2. Materials and methods

### 2.1. Animals

All animal experimental protocols were approved by the Animal Care Committee of School of Medicine, Shandong University. Male mice were used in our experiments.

*TPO-Cre* (thyroid peroxidase-Cre recombinase) mice were provided by the Laboratory of Metabolism, National Cancer Institute (USA). *Tshr^flox/flox^* (thyrotropin receptor locus of X-over P) mice were purchased from Guangzhou Cyagen biosciences. *FABP4-Cre* (fatty acid binding protein 4) mice were purchased from the Nanjing Model Animal Centre and were all controlled on a C57BL/6 J background. We generated *TPO-Cre/Tshr^flox/flox^* and *FABP4Cre/Tshr^flox/flox^* mice by crossing *Tshr^flox/flox^* mice with *TPO-Cre* or *FABP4-Cre* mice. *Tshr^±^*mice were purchased from Jackson Laboratory (USA) and had a 129S1/Sv background. Wild-type (*Tshr^+/+^)* mice and *Tshr-ko* (*Tshr^−/-^*) mice were obtained by intercrossing the *Tshr^±^*mice. PCR genotyping was carried out as described by the supplier. At 8 weeks of age, some of *TPO-Cre/Tshr^flox/flox^* mice were subcutaneously injected with 7 mU/g/d TSH or vehicle for 2 weeks before being sacrificed. Some *FABP4Cre/Tshr^flox/flox^* and *Tshr^−/-^* mice were exposed to cold (4°C) for 4 weeks before being sacrificed, and some of the *Tshr^−/-^* mice were fed a high-fat diet (purchased from Beijing Ke’ao Third Feed Co., Ltd.; the fat was 45% of Kcal diet).

Male C57BL/6J mice (8 weeks old) were obtained from Beijing Weitong Lihua Experimental Animal Technology Corporation (Beijing, China). SCH (subclinical hypothyroidism) mice were administered methimazole (MMI, 0.04 mg/kg/d) in the drinking water, and the control mice were administered a corresponding volume of vehicle (control group, n = 10) for 16 weeks as described before [12].

The main features of the mouse models used in our experiments were illustrated in supplementary Figure 1 (Supplementary Fig. S1).

The body composition of anesthetized animals were evaluated by Minispec-TD-NMR Analysers.

### 2.2. Cell culture

3T3-L1 cells were purchased from the American Type Culture Collection (ATCC). 3T3-L1 cells were cultured in DMEM (Gibco BRL, Gaithersburg, MD, USA) containing 10% FBS (foetal calf serum), 100 U/mL penicillin, and 0.1 mg/mL streptomycin (KeyGEN, Nanjing, China). To induce brown adipocyte differentiation, 3T3-L1 cells were cultured for 2 days with 0.5 mmol/L isobutylmethylxanthine (IBMX, Sigma-Aldrich), 1.0 μmmol/L dexamethasone (Sigma-Aldrich), 10 mmol/L insulin (Sigma-Aldrich), 1 nmol/L T3, 125 nmol/L indomethacin and 1 μmol/L rosiglitazone in DMEM supplemented with 10% FBS, then were treated for an additional 6 days with 10 mmol/L insulin, 1 nmol/L T3, 125 nmol/L indomethacin and 1 µmol/L rosiglitazone in DMEM containing 10% FBS. On day 9, approximately 90% of the cells differentiated into adipocytes.

Primary white fat stromal vascular fractions were obtained according to previously published methods [13]. In brief, epididymal and inguinal adipose tissues were minced and digested with 2.5 mg/ml of collagenase type I (Sigma) in PBS containing 0.25% pancreatin for 30–40 min at 38°C, and digestion was stopped with complete medium. Next, we filtered the cell suspensions with a 70 µm strainer, and after centrifuging and washing, the cells were finally dispersed and plated onto 10 cm dishes. The protocols for the culture and differentiation of SVF cells were the same as those used for 3T3-L1 cells.

Brown-differentiated 3T3-L1 adipocytes and primary SVF cells were starved in serum-free DMEM for 1 h before treatment. The cells were then stimulated with recombinant bovine TSH (bTSH, St. Louis, MO, USA, 10 mu/ml), TSHR siRNA (Oligo), AICAR (AMPK activator, 500 μM) and dorsomorphin (AMPK inhibitor, 4 μM) for 24 h according to the experimental design.

### 2.3. Temperature measurements

The body temperature of mice was measured at room temperature with a digital thermometer (TH-212 Microprobe-Thermometer; Beijing Hong’ou Cheng yun Co. Ltd, China).

### 2.4. Metabolic analysis

Mice were placed in metabolic cages (TSE Germany) to record their food intake, O_2_ consumption, CO_2_ production and physical activity. Energy expenditure (EE) was calculated using: kcal/h = 60* (0.003941* VO2 + 0.001106* VCO2) (Weir Equation).

### 2.5. OGTT (oral glucose tolerance test) and ITT (insulin tolerance test)

For the glucose tolerance test, mice were fasted for 14 h and administered 2 g/kg D-glucose. For the ITT, the mice were fasted for 6 h and intraperitoneally injected with recombinant human insulin (0.75 U/kg). Blood glucose levels were measured with a glucose metre using whole blood obtained from the mouse tail vein (Roche).

### 2.6. Serum FT4, FT3 and TSH measurements

Serum FT4 and FT3 levels were assessed with a radioimmunology kit (Jiuding Tianjin) and TSH levels were detected with a commercial ELISA kit.

### 2.7. Morphological studies

Tissues were fixed in 4% paraformaldehyde and embedded in paraffin. Haematoxylin and eosin staining was performed on multiple sections to observe morphological changes according to standard protocols. Immunohistochemical staining was performed on paraffin sections using anti-UCP1 primary antibodies (ProteinTech, 1:100). Immunofluorescence staining was conducted using a standard protocol with 1:100 and 1:1000 dilutions of the primary antibodies CD137 and MitoTracker Red (Invitrogen), respectively. Incubation was carried out in a humidifying chamber overnight at 4°C, then after which samples were incubated with the secondary antibodies for immunofluorescence staining. The cell nuclei were stained with DAPI (6-diamidino-2-phenylindole). Images were then captured using an Olympus BX51 system. All representative images shown in the figures represent repeats of at least three independent experiments.

### 2.8. Quantitative real-time PCR analysis

Total RNA was extracted using TRIzol reagent (Takara) according to the manufacturer’s instructions. Next, a Reverse Transcription System (Promega) was used to translate 1 μg of RNA into complementary DNA. Real-time PCR was carried out with the LC480 system (Roche) using SYBR Green Supermix (Takara). The primers used in the experiment are listed in Supplementary Table 1. Data were normalized to 36B4 and analysed by the ΔΔCT method.

### 2.9. Immunoblotting

Minced tissues were lysed in radioimmunoprecipitation assay (RIPA) buffer supplemented with protease and phosphatase inhibitors. SDS-PAGE was used to separate the protein lysates, which were then electroblotted onto polyvinylidene fluoride membranes (Millipore). The membranes were blocked and incubated with different primary antibodies overnight at 4°C, after which they were incubated with secondary antibodies for 1 h at room temperature. We used enhanced chemiluminescence reagents (Amersham Pharmacia) to perform western blot analyses according to the manufacturer’s protocol. The representative bands shown in the figures represent repeats with at least three mice. The antibodies used in this study are listed in Supplementary Table 2.

### 2.10. Statistical analysis

The data are presented as the means with standard errors of the mean (s.e.m.), and significant differences were analysed using two-tailed Student’s t-tests or 2-way ANOVA with Repeated Measures and Bonferonni post-hoc tests. Energy expenditure of all mouse models were analysed by analysis of covariance (ANCOVA) with fat mass and lean mass as two covariates in accordance with published methods [14].

## 3. Results

### 3.1. TSH promotes adiposity and metabolic disorders

Based on the characteristics of subclinical hypothyroidism (SCH), in which only serum TSH levels increased, we established the SCH mouse model. The SCH mice showed equal serum FT4/FT3 levels and slightly higher TSH levels than the normal controls (Supplementary Fig. S2 A, B and C).

To further confirm the direct effect of TSH on adiposity and eliminate the effect of methimazole, we established another mouse model with a thyroid-specific deletion of *Tshr* using the Cre-loxP recombination system. Expression of *Tshr* was lost in the thyroid of the *TPO-Cre*/*Tshr^flox/flox^* mice (abbreviated as *TPO-Tshr*), and the expression levels of *Tshr* in adipose tissue were normal (Supplementary Fig. S2D and E). *TPO-Tshr* mice were supplemented with L-thyroxine to maintain euthyroidism, and serum TSH levels did not differ between the *TPO-Tshr* mice and their wild-type littermates (Supplementary Fig. S2 F, G and H). At eight weeks of age, *TPO-Tshr* mice were subcutaneously injected with TSH or vehicle for 2 weeks. Correspondingly, serum TSH levels were increased nearly twofold in *TPO-Tshr* mice injected with TSH (Supplementary Fig. S2I), and no difference in serum FT4/FT3 levels was observed (Supplementary Fig. S2 J and K).

**Figure 1.**
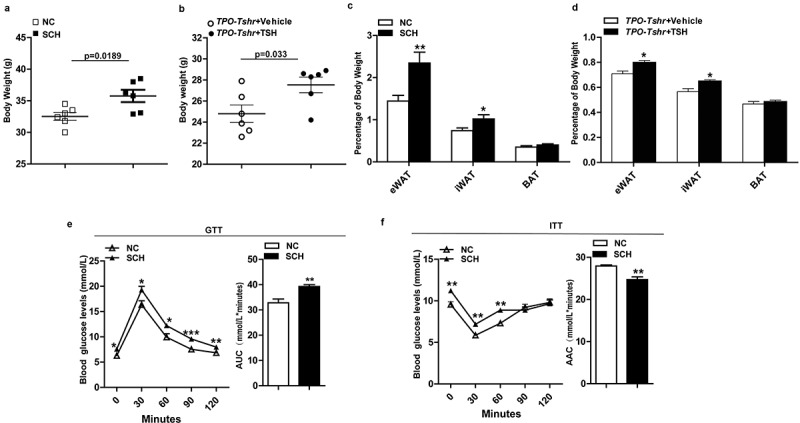
TSH promotes adiposity and its metabolic consequences. SCH: subclinical hypothyroidism mice; NC: normal control mice; *TPO-Tshr: TPO-Cre/Tshr^flox/flox^* mice; WT: wild-type littermates. AUC: area under curve; ACC: area above curve. (a) Body weight of the control and SCH mice (n = 10). (b) Body weight evaluation of the TPO-Tshr mice treated with TSH or vehicle for 2 weeks (n = 6). (c) Weight of eWAT, iWAT and BAT normalized to the body weights of the control and SCH mice (n = 8). (d) Weight of eWAT, iWAT and BAT normalized to the body weights of *TPO-Tshr* mice injected with TSH or vehicle (n = 6). (e, f) Results of the OGTT (f) and ITT (g) in the control and SCH mice (n = 8). The values represent the means ± s.e.m. Error bars represent s.e.m. Significant differences in SCH compared with NC mice and *TPO-Tshr* mice treated with TSH compared with vehicle controls are indicated by *P < 0.05, **P < 0.01 and ***P < 0.001 (Student’s t-test or 2-way ANOVA with repeated measures and Bonferroni post-hoc tests)

Higher body weights and an apparent increase of relative epididymal and inguinal WAT (eWAT and iWAT) weight were observed both in the SCH mice and *TPO-Tshr* mice injected with TSH fed a chow diet (CD), whereas the BAT weights were unaltered (Figure 1(a-d)). Excessive white fat deposits are more likely to accompany metabolic disturbances. As expected, SCH mice showed impaired glucose tolerance and insulin sensitivity when fed a CD (Figure 1(e,f)).

In our experiment, *TPO-Tshr* mice were injected with TSH for a short period, two weeks, whereas SCH mice were given methimazole for 16 weeks. The TSH levels of SCH mice were elevated for a long time. The different durations of TSH elevation may be the cause of the differences in weight gain.

These results demonstrate that elevated TSH increase body weight and worsen metabolic disorder profiles.

### 3.2. Elevated TSH levels decrease energy expenditure

**Figure 2.**
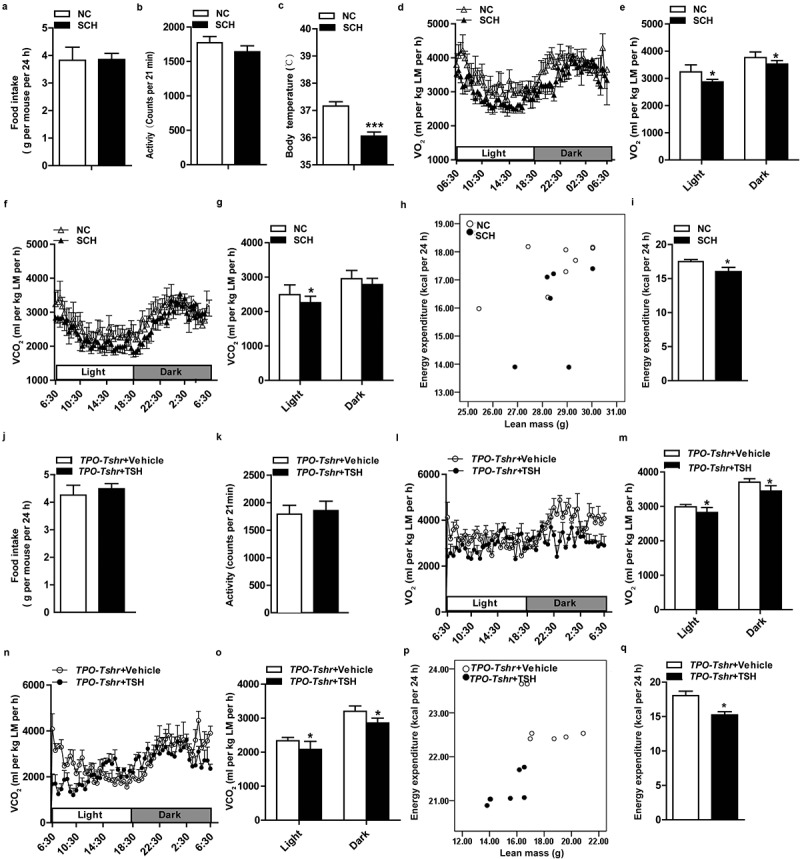
Elevated TSH levels reduces energy consumption

Body weight and adiposity are balanced by energy expenditure and caloric intake. Our data showed that there was no difference in food intake between the SCH mice and normal controls (Figure 2(a)). The physical activities of SCH mice were slightly decreased, but there was no significant difference between the two groups (Figure 2(b)). Lower body temperatures were observed in SCH mice (Figure 2(c)), and when normalized to lean mass, SCH mice showed lower O_2_ consumption and CO_2_ production (Figure 2(d-g)). As expected, energy expenditure was decreased in SCH mice as analysed by a statistical model, ANCOVA (analysis of covariance, both fat mass and lean mass were included in the model as covariates, P = 0.046; Figure 2(h,i)),These results demonstrated that SCH mice have lower metabolic rates, which may be caused by the elevated TSH levels.

SCH: subclinical hypothyroidism mice; NC: normal control mice; TPO-Tshr: *TPO-Cre/Tshr^flox/flox^* mice; WT: wild-type littermates; LM: lean mass. (A) The 24 h food intake of the control and SCH mice (n = 5–8). (B) Physical activity of the control and SCH mice over a 24 h recording period (n = 5–8). (C) Rectal temperature of the SCH mice and their controls measured at 16:00 (n = 5–8). (D-G) O_2_ consumption (D, E) and CO_2_ production (F, G) normalized to lean mass of the SCH and control mice were recorded in metabolic cages for 24 h (D, F: 24 h period; E, G: average of light and dark periods, respectively) (n = 5–8 for each group). (H, I) 24 h energy expenditure was compared between the control and SCH mice (H, energy expenditure per mouse was plotted against lean mass; I, the adjusted means of energy expenditure in the two groups analysed by ANCOVA with fat mass and lean mass as two covariates, P = 0.046). (J) The 24 h food intake of *TPO-Tshr* mice that had been treated with TSH or vehicle for 2 weeks (n = 5–8). (K) The physical activity of *TPO-Tshr* mice that had been treated with TSH or vehicle for 2 weeks was recorded for 24 h (n = 5). (L-O) O_2_ consumption (L, M) and CO_2_ production (N, O) normalized to lean mass of the *TPO-Tshr* mice treated with TSH or vehicle for 2 weeks were recorded in metabolic cages for 24 h (L and N: 24 h period; M and O: average of light and dark periods, respectively) (n = 5–7 for each group). (P,Q) 24 h energy expenditure was compared between the *TPO-Tshr* mice treated with TSH and vehicle (P, energy expenditure per mouse was plotted against lean mass; I, the adjusted means of energy expenditure in the two groups analysed by ANCOVA with fat mass and lean mass as two covariates, P = 0.049).Values represent the means ± s.e.m. Error bars represent s.e.m. Significant differences in SCH mice compared with NC or *TPO-Tshr* mice treated with TSH compared with vehicle controls are indicated by *P < 0.05, **P < 0.01 and ***P < 0.001 (Student’s t-test or analysis of covariance (ANCOVA)).

To further validate the above results and exclude the possible influence of methimazole on metabolism, we performed metabolic cage analyses on thyroid-specific knockout of *Tshr* mice (*TPO-Tshr* mice). As shown in supplementary Fig. S3, no differences in O_2_ consumption, CO_2_, energy expenditure and physical activity were observed between the *TPO-Tshr* mice and their wild-type littermates (supplementary Fig. S3A-D). At age of 8 weeks old, *TPO-Tshr* mice were injected with TSH and vehicle, respectively, for two weeks. As expected, the TSH levels increased significantly after TSH injection. We next compared the metabolic status of the two groups, observing that *TPO-Tshr* mice injected with TSH also had lower O_2_ consumption and CO_2_ production normalized to lean mass (Figure 2(l-o)), energy expenditure was also decreased in *TPO-Tshr* mice injected with TSH mice as analysed by ANCOVA (both fat mass and lean mass were included in the model as covariates, P = 0.049; Figure 2(p,q)),whereas the food intake and physical activities of these mice were not different (Figure 2(j,k)).

Taken together, these results demonstrated that elevated TSH levels promote adiposity by reducing the energy expenditure.

### 3.3. Tshr*-knockout mice resist adiposity and associated metabolic complications*

TSH works by binding to its receptor (*Tshr*). To further investigate the effects of TSH on obesity and energy metabolism, we generated global *Tshr-*knockout mice (abbreviated as *Tshr*^−/-^). These mice were fed a levothyroxine-supplemented diet to maintain normal serum TSH and FT4 levels (supplementary Fig. S4A and B). To exclude other influences caused by global *Tshr* deficiency in the embryonic stage, we also established an adipose-specific *Tshr* deletion mouse model. The expression of *Tshr* in adipose was lost in the *FABP4-Cre*/*Tshr^flox/flox^* mice (abbreviated as *FABP4-Tshr*), whereas the expression of *Tshr* in the thyroid gland was normal (supplementary Fig. S4 C and D) and no differences in FT4 and FT3 levels were observed between the two groups (supplementary Fig. S4E and F).

**Figure 3.**
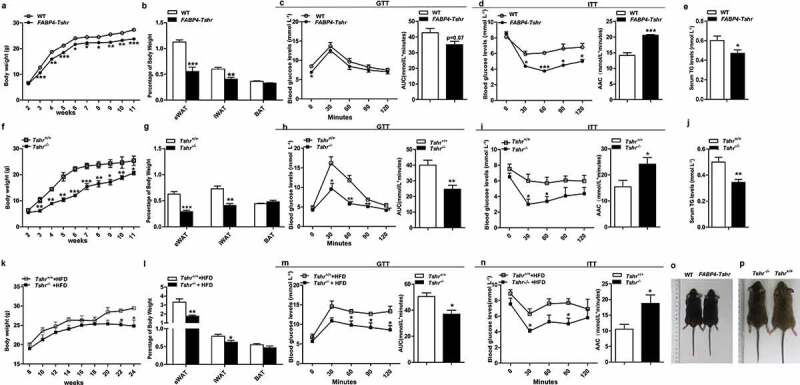
*Tshr*-knockout mice resist adiposity and its metabolic complications

We observed that *FABP4-Tshr* mice exhibited lower body weights from 3 weeks of age to adulthood than the wild-type mice when fed a chow diet (CD) (Figure 3(a)). Furthermore, *FABP4-Tshr* mice had a remarkably decreased white fat content, with the relative eWAT and iWAT weights being significantly reduced, whereas the BAT weight was unchanged (Figure 3(b)). In line with their lean phenotype, *FABP4-Tshr* mice have a tendency to improve fasting glucose but it did not reach statistical significance (Figure 3(c)), meanwhile they showed improved insulin tolerance (Figure 3(d)) and lower TG levels (Figure 3(e)) when fed a CD.

*FABP4-Tshr: FABP4-Cre/Tshr^flox/flox^* mice; WT: wild-type littermates; *Tshr^−/-^*: global Tshr-knockout mice; *Tshr^+/+^*: the wild-type littermates of *Tshr^−/-^* mice. AUC: area under curve; ACC: area above curve. (A) Body weights of the *FABP4-Tshr* mice and their wild-type littermates fed the CD were recorded for 11 weeks (n = 6 for each group). (B) Weights of the eWAT, iWAT and BAT normalized to body weights of the *FABP4-Tshr* mice and the wild-type fed the CD (n = 6 for each group). (C, D) Results of the OGTT (C) and ITT (D) for *FABP4-Tshr* mice and wild-type mice fed the CD (n = 6 for each group). (E) Serum triglyceride levels in *FABP4-Tshr* mice and wild-type fed the CD. (n = 10) (F) The body weights of the *Tshr^−/-^* and wild-type mice fed the CD were recorded for 11 weeks (n = 6 for each group). (G) Weights of eWAT, iWAT and BAT normalized to the body weights of the *Tshr^−/ –^* and wild-type mice fed the CD (n = 8 for each group). (H, I) Results of the OGTT (H) and ITT (I) for *Tshr^−/-^* and wild-type mice fed the CD (n = 6 for each group). (J) Serum triglyceride levels in the *Tshr^−/-^* and wild-type mice fed the CD (n = 9). (K) Weight gain of the *Tshr^−/-^* mice and their wild-type littermates fed a high-fat diet (HFD) for 16 weeks (n = 7–8 for each group). (L) Weights of eWAT, iWAT and BAT normalized to the body weights of the *Tshr^−/-^* and wild-type mice fed the HFD (n = 7–8 for each group). (M, N) Results of the OGTT (M) and ITT (N) for the *Tshr^−/-^* and wild-type mice fed the HFD (n = 7–8 for each group). (O, P) Representative photograph of 12-week-old *Tshr^−/-^* mice, *FABP4-Tshr* mice and their wild-type littermates. Values represent the means±s.e.m. Error bars represent s.e.m. Significant differences in *FABP4-Tshr* mice compared with their wild-type littermates or *Tshr^−/-^* mice compared with *Tshr^+/+^* are indicated by *P < 0.05, **P < 0.01 and ***P < 0.001 (Student’s t-test or 2-way ANOVA with repeated measures and Bonferroni post-hoc tests).

Consistent with the *FABP4-Tshr* mice, *Tshr^−/-^* mice also showed markedly decreased body weights and the relative eWAT and iWAT content whereas the BAT weights were unaltered (figure 3(f,g)) compared with those of the wild-type mice. *Tshr^−/-^* mice also exhibited improved glucose tolerance (Figure 3(h)), insulin tolerance (Figure 3(i)) and lower TG levels (Figure 3(j)).

We next determined the effects of *Tshr* knockout on high-fat diet (HFD)-induced adiposity. The serum FT4 levels were not different between the two groups (supplementary Fig. S4G). As shown in Figure 3(k), *Tshr^−/-^* mice were resistant to HFD-induced adiposity (Figure 3(k)), no marked change was observed in the brown fat, the epididymal fat mass was significantly lower than that of the wild-type group fed HFD, while the inguinal subcutaneous fat mass was slightly lower, which most likely because HFD has a greater influence on the visceral adipose tissues than subcutaneous tissues [15]. Consistent with these results, when fed HFD, *Tshr^−/-^* mice also showed improved glucose tolerance and insulin sensitivity (Figure 3(m,n)). Figure 3(o,p) present representative photographs of 12-week-old *Tshr^−/-^* mice, *FABP4-Tshr* mice and their wild-type littermates.

Based on these results, we conclude that *Tshr* knockout potently protects mice from diet-induced adiposity and metabolic disorders.

### 3.4. Tshr *knockout increases energy expenditure*

**Figure 4.**
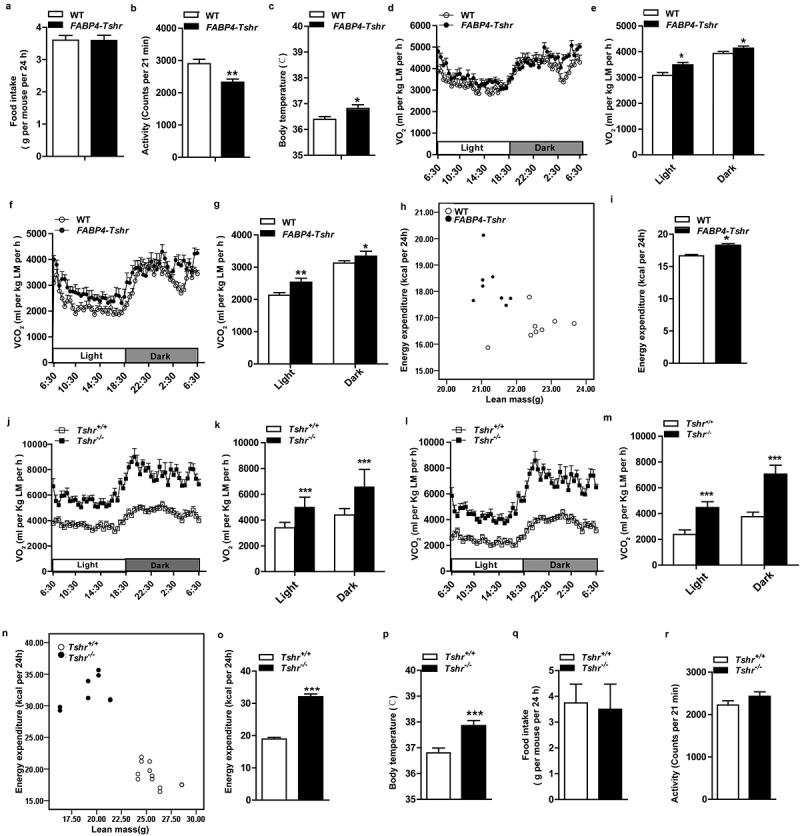
*Tshr* knockout increases energy expenditure

*FABP4-Tshr* mice fed a CD exhibited a lean phenotype compared to their wild-type littermates, but the decrease in adiposity was not due to reduced food intake (Figure 4(a)) and their physical activity was significantly decreased (Figure 4(b)). Additionally, we observed that *FABP4-Tshr* mice had a higher body temperature (Figure 4(c)). In contrast to the *TPO-Tshr*mice injected with TSH and SCH mice, *FABP4-Tshr* mice showed the following characteristics of higher energy expenditure: higher O_2_ consumption (Figure 4(d,e)) and CO_2_ production (figure 4(f,g)) normalized to lean mass, and the 24 h energy expenditure of *FABP4-Tshr* mice was increased as analysed by ANCOVA (both fat mass and lean mass were included in the model as covariates, P = 0.015; Figure 4(h,i)).

*FABP4-Tshr: FABP4-Cre*/*Tshr^flox/flox^* mice; WT: wild-type littermates; *Tshr*^−/-^: global *Tshr-*knockout mice; *Tshr^+/+^*: the wild-type littermates of *Tshr*^−/-^ mice; LM: lean mass. (A) The 24 h food intake was measured in both *FABP4-Tshr* mice and their wild-type littermates (n = 7–8 for each group). (B) Physical activity was recorded for the *FABP4-Tshr* mice and their wild-type littermates over 24 h (n = 7–8). (C) Rectal temperatures of the *FABP4-Tshr*mice and their wild-type littermates (n = 6–8). (D-G) O_2_ consumption (D, E) and CO_2_ production (F, G) normalized to lean mass of the *FABP4-Tshr*mice and their wild-type littermates were recorded in metabolic cages for 24 h (D and F: 24 h period; E and G: average of light and dark periods) (n = 7–8). (H,I) 24 h energy expenditure was compared between *FABP4-Tshr* mice and their wild-type littermates (H, energy expenditure per mouse was plotted against lean mass; I, the adjusted means of energy expenditure in the two groups analysed by ANCOVA with fat mass and lean mass as two covariates, P = 0.015). (J-M) O_2_ consumption (J, K) and CO_2_ production (L, M) normalized to lean mass of the *Tshr^−/-^* mice and their wild-type littermates during a 24 h period (J and L: 24 h period; K and M:average of light and dark periods) (n = 6–8 for each group). (N,O) 24 h energy expenditure was compared between the *Tshr^−/-^* mice and their wild-type littermates (N, energy expenditure per mouse was plotted against lean mass; O, the adjusted means of energy expenditure in the two groups analysed by ANCOVA with fat mass and lean mass as two covariates, P = 0.006). (P) Comparison of the body temperatures of the *Tshr^−/-^* and wild-type mice (n = 7). (Q) The 24 h food intake of the *Tshr^−/-^* and the wild-type mice (n = 6). (R) Physical activity of the *Tshr^−/-^* mice and their wild-type littermates in a 24 h recording period (n = 6). Values represent the means±s.e.m. Error bars represent s.e.m. Significant differences in *FABP4-Tshr*mice compared with their wild-type littermates or *Tshr^−/-^* mice compared with *Tshr^+/+^* are indicated by *P < 0.05, **P < 0.01 and ***P < 0.001 (Student’s t-test or analysis of covariance (ANCOVA)).

We further analysed the metabolic rates of the *Tshr^−/-^* mice, and consistent with the *FABP4-Tshr* mice, energy expenditure was greatly increased in *Tshr^−/-^* mice, which was confirmed by increased O_2_ consumption (Figure 4(j,k)) and CO_2_ production (Figure 4(l,m)) when normalized to lean mass, the 24 h energy expenditure of *Tshr^−/-^* mice was also significantly increased as analysed by ANCOVA (fat mass and lean mass were included in the model as covariates, p = 0.006, Figure 4(n,o)), along with elevated body temperature (Figure 4(p)). However, 24 h food intake and physical activity were not different between the two groups (Figure 4(q,r)).

Interestingly, our results showed that the metabolic rates of *FABP4-Tshr* mice were marginally improved compared to their wild-type littermates. However, the metabolic rates of *Tshr^−/-^* mice were considerably higher, and this phenomenon was probably caused by the different strains of the mice. The background of the *Tshr^−/-^* mice was 129S6/SvEvTac (129), and the background of the *FABP4-Tshr* mice was C57BL/6 (B6). Katrine Almind et al. [16] observed that large numbers of brown adipocytes were distributed in the peri-muscular and intermuscular adipose tissue of the legs of 129 mice. It is exactly this ‘ectopic brown fat’ that resulted in a higher metabolism in 129 mice than in B6 mice.

These results suggest that *Tshr* knockout protects mice from obesity by increasing energy expenditure.

### 3.5. Tshr *knockout induces a white-to-brown fat transition*

BAT is characterized by increased energy metabolism via thermogenesis. However, the histological morphology of BAT showed the presence of increased numbers of lipid droplets (supplementary Fig. S5A). The expression of related thermogenic genes and UCP1 protein levels were decreased in both *FABP4-Tshr* and *Tshr^−/-^* mice (Supplementary Fig. S5B-E), indicating the decreased thermogenesis of BAT. However, the expression of ASC1, a white adipocyte marker, did not increase in BAT in *Tshr*- knockout mice (supplementary Fig.S5 F).

**Figure 5.**
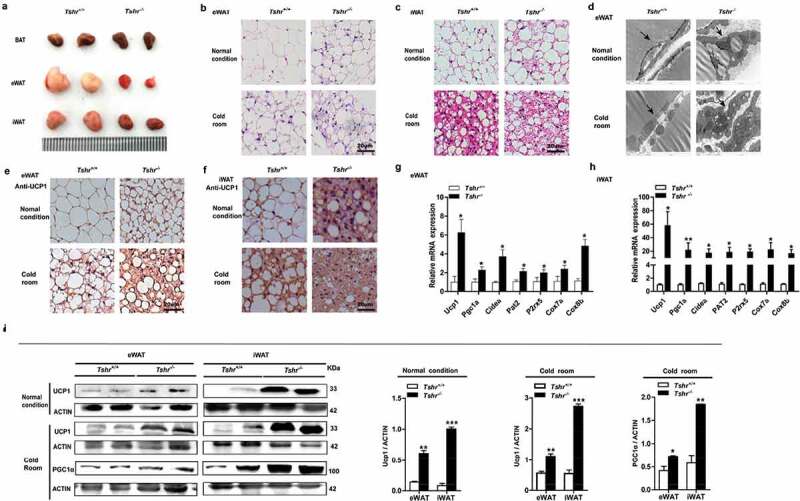
*Tshr* knockout induces the white-to-brown fat transition in *Tshr^−/-^* mice

Compared with the wild-type mice, *Tshr^−/-^* mice showed a reduced WAT content with a beige colour (Figure 5(a)). HE staining showed that the eWAT and iWAT of *Tshr^−/-^* mice contained brown adipocyte-like cells that were filled with multilocular lipid droplets (Figure 5(b,c)), and electron microscopy showed smaller, multilocular adipocytes that contained increased numbers of mitochondria (Figure 5(d)) in the eWAT of *Tshr^−/-^* mice. This morphological transition appeared in both the eWAT and iWAT, and the transition was more obvious when the mice were subjected to cold stress. Consistently, the BAT-specific marker UCP1 showed markedly positive staining in the eWAT and iWAT of *Tshr^−/-^* mice (Figure 5(e,f)). In addition, other thermogenic genes, including peroxisome proliferator-activated receptor gamma coactivator 1α (PGC1α), cell death-inducing DFFA-like effector a (Cidea), cytochrome oxidase 7a (Cox7a), and cytochrome oxidase 8b (Cox8b) as well as beige cell markers, such as proton assistant amino acid transporter-2 (PAT2) and purinergic receptor P2X ligand-gated ion channel 5 (P2rx5), were significantly increased in both the eWAT and iWAT of *Tshr^−/-^* mice (Figure 5(g,h)). As shown in western blots, the levels of UCP1 and PGC-1α proteins were significantly increased, particularly in response to cold stimulation (Figure 5(i)). Our results demonstrated that the browning of inguinal subcutaneous fat is more notable than in epididymal fat.

*Tshr^−/-^*: global Tshr-knockout mice; *Tshr^+/+^*: the wild-type littermates of *Tshr^−/-^* mice. (A) Representative images of the BAT, eWAT and iWAT depots in wild-type and *Tshr^−/-^* mice. (B, C) Representative HE staining of eWAT and iWAT of *Tshr^−/-^* and wild-type mice housed at room temperature and in a cold room (4°C) for 4 weeks, respectively (scale bar, 20 μm). (D) Electron microscopy of eWAT in wild-type and *Tshr^−/-^* mice under normal conditions and after four weeks of cold stress (scale bar 1 μm), the arrows refer to lipid droplets and mitochondria. (E, F) Immunohistochemical staining of UCP1 in eWAT and iWAT of wild-type and *Tshr^−/-^* mice housed at room temperature or 4°C for 4 weeks, respectively (scale bar 20 μm). (G, H) Real-time PCR analysis of the expression of thermogenic and related genes is shown in eWAT and iWAT of *Tshr^−/-^*and their wild-type littermates (n = 6). (I) Representative western blots showing the key protein levels of UCP1 and PGC-1α in the eWAT and iWAT of wild-type and *Tshr^−/-^* mice housed under different conditions. Actin was used as the loading control. Values represent the means±s.e.m. Error bars represent the s.e.m. Significant differences in *Tshr^−/-^* mice compared with *Tshr^+/+^* are indicated by *P < 0.05, **P < 0.01 and ***P < 0.001 (Student’s t-test).

**Figure 6.**
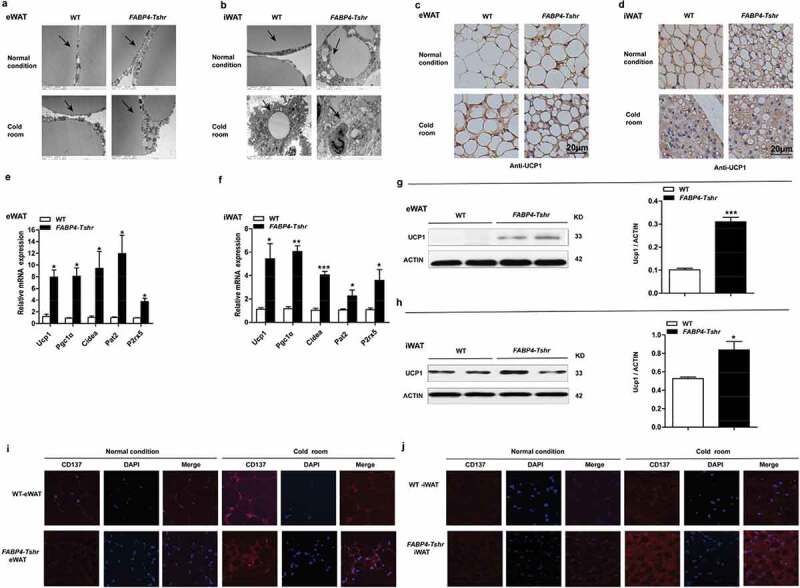
*Tshr* knockout induces the white-to-brown fat transition in *FABP4-Tshr* mice

In accordance with the *Tshr^−/-^* mice, the *FABP4-Tshr* mice also exhibited smaller lipid droplets and increased mitochondria (Figure 6(a,b)). Furthermore, immunohistochemical staining of UCP1 increased significantly (Figure 6(c,d)), and the expression of thermogenic genes and the beige cell markers PAT2 and P2rx5 was also remarkably increased in both the eWAT and iWAT of *FABP4-Tshr* mice (Figure 6(e,f)). Accordingly, western blotting showed that the protein levels of UCP1 in eWAT and iWAT were also increased (Figure 6(g,h)), and CD137 (characteristically expressed in beige cells) was expressed at significantly higher levels than in wild-type mice at both room temperature and 4°C (Figure 6(i,j)). This phenomenon was more obvious under exposure to cold stress.

*FABP4-Tshr: FABP4-Cre/Tshr^flox/flox^* mice; WT: wild-type littermates. (A, B) Electron microscopy of eWAT (A) and iWAT (B) in *FABP4-Tshr* mice and their wild-type littermates under normal conditions and after four weeks of cold stress (scale bar 2 μm); the arrows refer to lipid droplets and mitochondria. (C, D) Immunohistochemical staining of UCP1 in eWAT (C) and iWAT (D) of *FABP4-Tshr* mice and their wild-type littermates housed at room temperature or 4°C for 4 weeks (scale bar 20 μm). (E, F) Real-time PCR analysis of the expression of thermogenic and related genes in eWAT (E) and iWAT (F) of *FABP4-Tshr* and wild-type mice (n = 6). (G, H) Representative western blots showing the key protein levels of UCP1 in the eWAT and iWAT of *FABP4-Tshr* and wild-type mice housed under normal conditions. (I, J) Immunofluorescence staining of CD137 in eWAT and iWAT of *FABP4-Tshr* mice and their wild-type littermates housed at room temperature (q) and 4°C (r) for 4 weeks, respectively (scale bar 20 μm). Values represent the means±s.e.m. Error bars represent the s.e.m. Significant differences in *FABP4-Tshr* mice compared with their wild-type littermates are indicated by *P < 0.05, **P < 0.01 and ***P < 0.001 (Student’s t-test).

Based on these results, *Tshr* knockout activates functional brown-like (beige) adipocytes in both eWAT and iWAT depots, leading to increased energy expenditure.

### 3.6. Tshr *knockout induces the browning of WAT via AMPK/PRDM16/PGC1a*

**Figure 7.**
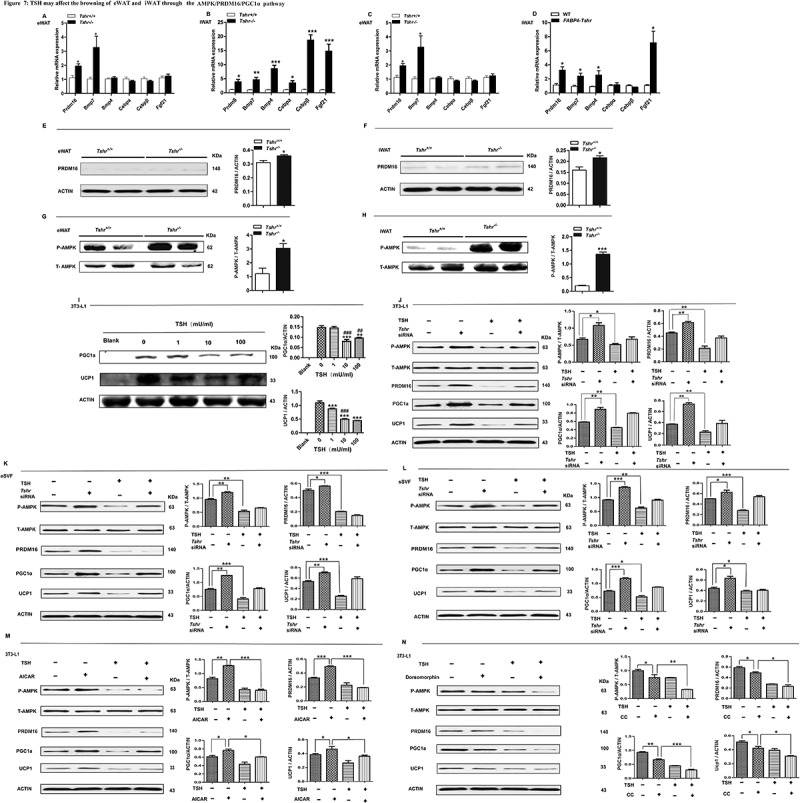
TSH may affect the browning of eWAT and iWAT through the AMPK/PRDM16/PGC1α pathway

Our previous results confirmed that *Tshr* knockout induces the browning of white fat. To explore the potential mechanism of TSH in regulating the browning of WAT, we screened a panel of genes involved in brown or beige fat development in the WAT of *Tshr^−/-^* and *FABP4-Tshr* mice. The gene expression and protein levels of PR domain-containing 16 (PRDM16) (Figure 7(a-d)) were increased in the eWAT and iWAT of both genotypes (Figure 7(e,f)). Furthermore, we observed that AMPK phosphorylation was notably elevated in the eWAT of *Tshr^−/-^* mice (Figure 7(g,h)). Recent studies have shown that AMPKa1 (PRKAA1) mediates DNA demethylation at the PRDM16 promoter during BAT development and regulates brown adipogenesis [17]. PRDM16 could promote UCP1 expression by activating PGC1α, and we observed that PGC1α was increased in both the eWAT and iWAT of *Tshr^−/-^* mice. According to these results, we propose that TSH regulates the browning of eWAT and iWAT, probably by the common pathway of AMPK/PRDM16/PGC1α.

*FABP4-Tshr: FABP4-Cre/Tshr^flox/flox^* mice; WT: wild-type littermates; *Tshr^−/-^*: global TSHR knockout mice; *Tshr^+/+^*: the wild type littermates of *Tshr^−/-^* mice. (A, B) Expression profiles of genes related to the browning of WAT in the eWAT and iWAT of wild-type and *Tshr^−/-^* mice (n = 6. (C, D) Gene expression profiles related to the browning of WAT in the eWAT and iWAT of wild-type and *FABP4-Tshr* mice(n = 6). (E, F) Levels of the PRDM16 protein in the eWAT and iWAT of wild-type and *Tshr^−/-^* mice. (G, H) Levels of the AMPK protein in the eWAT and iWAT of *Tshr^−/-^* and wild-type mice housed under normal conditions. (I) The dose-dependent effects of TSH on PGC1α and UCP1 protein expression in brown-differentiated 3T3-L1 cells. (J-L) Brown-differentiated 3T3-L1 cells and primary SVF cells were transfected with non-targeting or *Tshr* siRNA. After 24 h, the cells were incubated with or without TSH (10 mu/ml) for 24 h. Total AMPK, p-AMPK, PRDM16, PGC1α and UCP1 expression were detected by western blotting. (M, N) Brown-differentiated 3T3-L1 cells were incubated with or without AICAR/Dorsomorphin and TSH (10 mu/ml) for 24 h. Total AMPK, p-AMPK, PRDM16, PGC1α and UCP1 expression were detected by western blotting. Values represent the means±s.e.m. Error bars represent s.e.m. Significant differences in *FABP4-Tshr* mice compared with their wild-type littermates, *Tshr^−/-^* mice compared with *Tshr^+/+^*,TSH(1, 10, 100MU/ml) compared with TSH (0MU/ml), non-TSH group compared with TSH group. TSH(10mU/ml, 100mU/ml) compared with TSH(1mU/ml) were indicated by ^#^P < 0.05, ^##^P < 0.01 and ^###^P < 0.001.

We further conducted in vitro experiments to validate our hypothesis. Our data showed that the expression of UCP1 in brown-differentiated 3T3-L1 cells decreased in a dose-dependent manner following TSH treatment (Figure 7(i)). Furthermore, we applied siRNA-mediated depletion of *Tshr* and TSH stimulation to show that TSH specifically inhibited p-AMPK, PRDM16, PGC1α and UCP1 expression in brown-differentiated 3T3-L1 cells and primary SVF cells (from the eWAT and iWAT) (Figure 7(j-l)). To reconfirm that *Tshr* knockout-induced browning is mediated by AMPK, we treated brown-differentiated 3T3-L1 adipocytes with either an activator of AMPK (AICAR) or an inhibitor of AMPK (Dorsomorphin) to increase or reduce AMPK activity. Our data showed that TSH significantly blocked AICAR stimulation of PRDM16, PGC1α and UCP1 expression, whereas TSH enhanced the effects of dorsomorphin (Figure 7(m,n)).

These results demonstrate that *Tshr*-knockout induction of the beige adipocyte differentiation in WAT may be caused by activating AMPK/PRDM16/PGC1α.

## 4. Discussion

In this study, we generated SCH, *TPO-Cre/Tshr^flox/flox^, FABP4 Cre/Tshr^flox/flo^*, and *Tshr^−/-^* mouse models to directly identify a role for TSH in WAT browning and energy expenditure. We provided evidence that elevated TSH promotes obesity by decreasing energy consumption and that the knockout of *Tshr* induces browning of WAT, leading to increased energy expenditure and resistance to metabolic disorders. Our additional in vitro experiments demonstrated that AMPK/PRDM16/PGC1α may be a signalling pathway for TSH in the regulation of the browning of white fat. Our study reveals that TSH inhibits the browning of white fat and decrease energy expenditure, which may be one of the mechanisms by which TSH promotes adiposity.

TSH has been reported to promote thermogenesis in BAT by increasing the activity of type II iodothyronine deiodinase (DIO2) and activating the p38 (MAPK)/ERK pathway, protecting the organism from hypothermia in a severe hypothyroid state [18–20]. BAT activation can limit weight gain, improve insulin resistance and corrects hyperlipidaemia in mice [6], transplantation of BAT causes resistance to obesity and its metabolic consequence [21,22]. According to some retrospective studies, the prevalence of BAT is lower in patients with a higher BMI [23], and the preponderance and activity of cold-activated BAT decreases with increasing adiposity in healthy participants [24,25]. However, many studies have reported a positive relationship between TSH and obesity or metabolic disorders [8–11]. In our study elevated TSH levels (SCH or *TPO-Tshr*mice injected with TSH) exhibited decreased energy expenditure and greater fat deposition. Correspondingly, *Tshr*-knockout mice showed higher metabolic rates and a lean phenotype despite their decreased brown fat thermogenesis. These results suggest that TSH may exert other effects to counteract the benefits of brown fat activation.

There are two distinct types of brown adipocytes, the classical brown adipocytes (constitutive BAT), which originate from the embryo and are located in the interscapular region of mice, and beige or brite cells, which are interspersed within WAT and skeletal muscle [26,27] and are also known as recruitable BAT. Recruitable BAT can be promoted by some stimuli, such as prolonged cold stress or β-adrenergic receptor activation [28,29]. Browning is a process that is characterized by the inducement of UCP1-positive multilocular adipocytes (beige adipocytes) and can increase the thermogenic capacity of the organism. Beige cells arise directly from preadipocytes that are derived from mesodermal stem cells [30] or from white adipocytes through trans-differentiation [31,32]. These two independent processes might coexist during the browning of adipocytes.

Our results showed that *Tshr*-knockout mice acquired characteristics of a beige phenotype in both iWAT and eWAT, including changes in morphology, increased expression of genes and proteins related to the differentiation/recruitment of beige adipocytes, increased expression of UCP1 and beige adipocyte markers, such as PAT2, P2RX5, CD137 [33,34], and finally increased energy dissipation.

Furthermore, the browning extent of iWAT was more significant than eWAT in *Tshr-*knockout mice, particularly when the mice were exposed to cold stress. The propensity for beige cell differentiation might differ between these two WAT deposits. Although beige adipocytes can be induced in epididymal white fat that is stimulated by strong β adrenergic agonist, some research has shown that the browning capacity of visceral fat is much lower than subcutaneous fat [35].

*Tshr* knockout seemed to induce the expression of many genes associated with brown/beige development and function. PGC-1α is highly expressed in BAT but is expressed at low levels in WAT [36]. Overexpression of PGC1α in WAT induces the browning of WAT, increasing the expression of mitochondrial and thermogenic genes [37,38]. Our results showed that PGC1α expression increased significantly in both eWAT and iWAT, supporting that PGC-1α might be critical for the thermogenic effects caused by *Tshr* knockout.

We further observed that PRDM16 expression levels increased in both eWAT and iWAT. PRDM16 is highly expressed in brown adipocytes compared to white adipocytes. When PRDM16 was overexpressed in white fat cell progenitors, these cells finally showed a brown adipocyte phenotype. Transgenic expression of PRDM16 in white fat depots at physiological levels stimulates the formation of brown adipocytes. PRDM16 activates brown fat cell features by activating PGC-1α through direct protein binding [39]. Therefore, we identified PRDM16 as a common upstream key regulator of WAT browning in *Tshr^−/-^* mice.

AMPK is a key regulator of energy metabolism and plays a crucial role in both cellular and whole-body energy status [40,41]. Obesity is well known to suppress AMPK activity [42]. AMPK activation induces an accumulation of brown-like adipocytes in the eWAT of mice, and AMPK directly phosphorylates PGC-1α and increases mitochondrial biogenesis [43]. According to recent research, AMPKa1 (PRKAA1) mediates DNA demethylation at the PRDM16 promoter during BAT development and regulates brown adipogenesis [17]. The levels of p-AMPK were increased in both the iWAT and eWAT of *Tshr^−/-^* mice, and our in vitro experiments showed that AICAR significantly blocked the TSH-inhibited UCP1 and PGC1α expression, and dorsomorphin enhanced the effects of TSH. These results suggest that the AMPK/PRDM16/PGC-1α pathway may be involved in the browning induced by *Tshr* knockout.

AMPK is also abundant in BAT and the brain. BAT activity and energy expenditure are increased either by suppression of AMPK activity in the hypothalamus or by activation of AMPK in BAT [44]. The mechanism underlying *Tshr* knockout inhibition of BAT function requires further investigation.

Other factors that regulate brown adipocyte differentiation, such as FGF21 [45], BMP7 [46], BMP4 [47], CCAAT/enhancer-binding protein-α (CEBP-α) [48] and CEBP-β [49] were also increased in the WAT of *FABP4-Tshr* and *Tshr^−/-^* mice. However, the expression levels of these genes in the two types of WAT were not consistent. Additional fat-mapping experiments are needed to resolve this question.

TSH consists of a common α-subunit and a unique-β subunit, the latter is responsible for hormone specificity. TSH is produced by the anterior pituitary and the pituitary is believed to be the only source of TSH used by the thyroid currently. In 2009, Jeremy S et al found that a new TSHβ isoform is expressed in peripheral blood leukocytes (PBL) and the thyroid [50]. The recent research published by Ferran Comas et al found that TSHβ gene also expressed in adipose tissue and both visceral and subcutaneous adipose tissue TSHβ gene expression was positively correlated with the expression of mitochondrial function of adipocytes, the obese patients with bariatric surgery-induced weight loss resulted in increased subcutaneous adipose tissue TSHβ gene expression in parallel to increased Pgc1α. However their results showed that adipose TSHβ expression was not related with BMI and no significant associations were observed between adipose tissue TSHβ gene expression and browning [51]. It seems that the results were not consistent with our and other’s results [8–11]. Here we studied the effect of circulation TSH and TSHR knockout on the browning of white fat, we used two mice models of TSH elevation and two TSHR-knockout models to confirm our conclusion. Whether the expression of TSH β gene expression in adipose tissue is exactly the same as that in circulation and its specific roles needs to be further verified.

In summary, TSH inhibits the browning of white fat and decreases energy expenditure, thus potentially contributing to the pathogenesis of adiposity. We have revealed that AMPK/PRDM16/PGC1α may be the mechanism by which TSH regulates the transcription of UCP1. These findings reveal an important role of TSH in regulating the browning of WAT and in energy metabolism.

## Supplementary Material

Supplemental Material
