# Supplementary material for: TSH promotes adiposity by inhibiting the browning of white fat
Source: Adipocyte. 2020 Jun 24;9(1):264–78. doi: 10.1080/21623945.2020.1783101 (PMC7469524; doi:10.1080/21623945.2020.1783101)

Supplentmary Figure 1:The mouse models and their main features

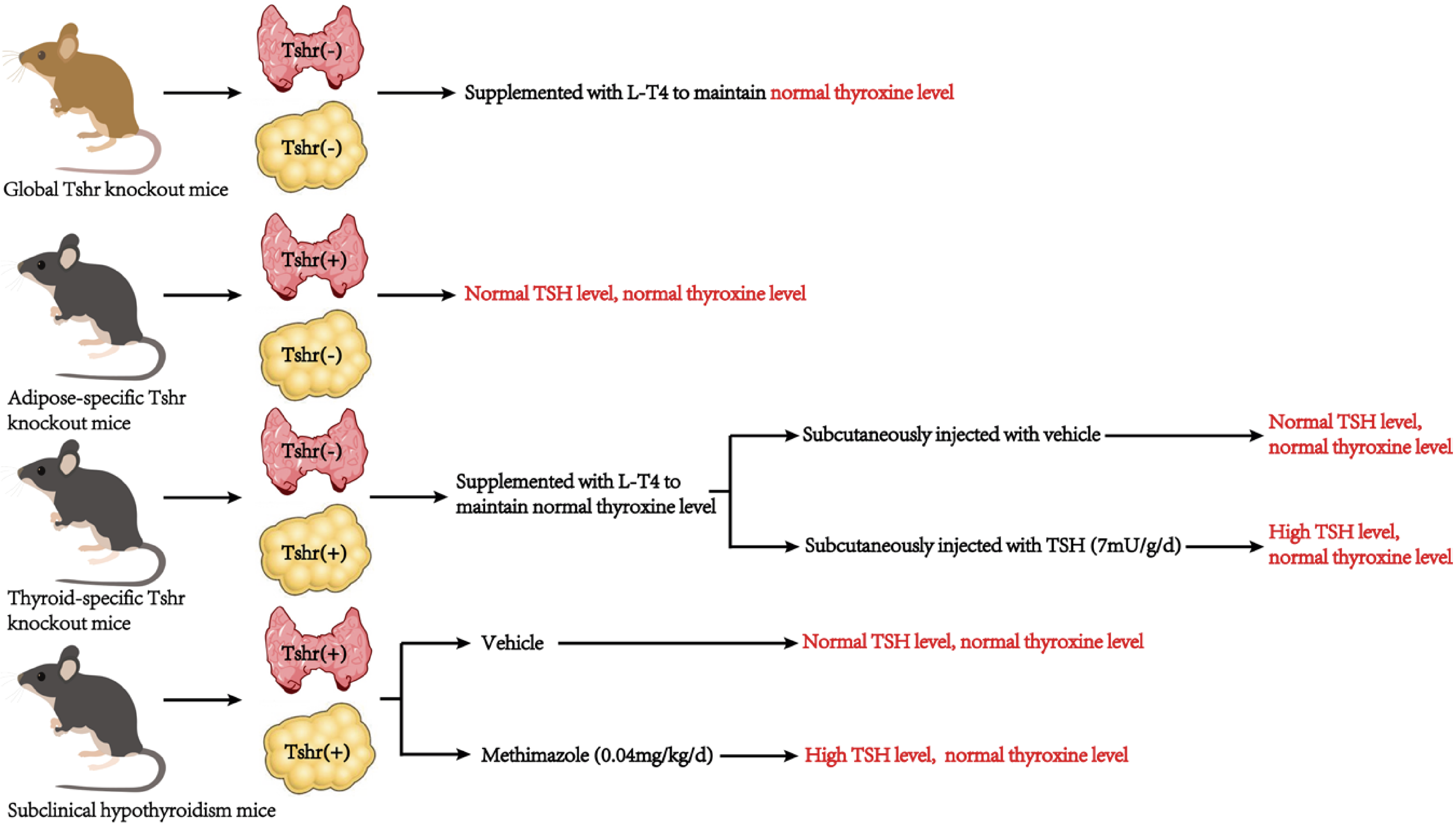

Supplementary Figure S2: Evaluation of the SCH and TPO-Tshr mouse models.

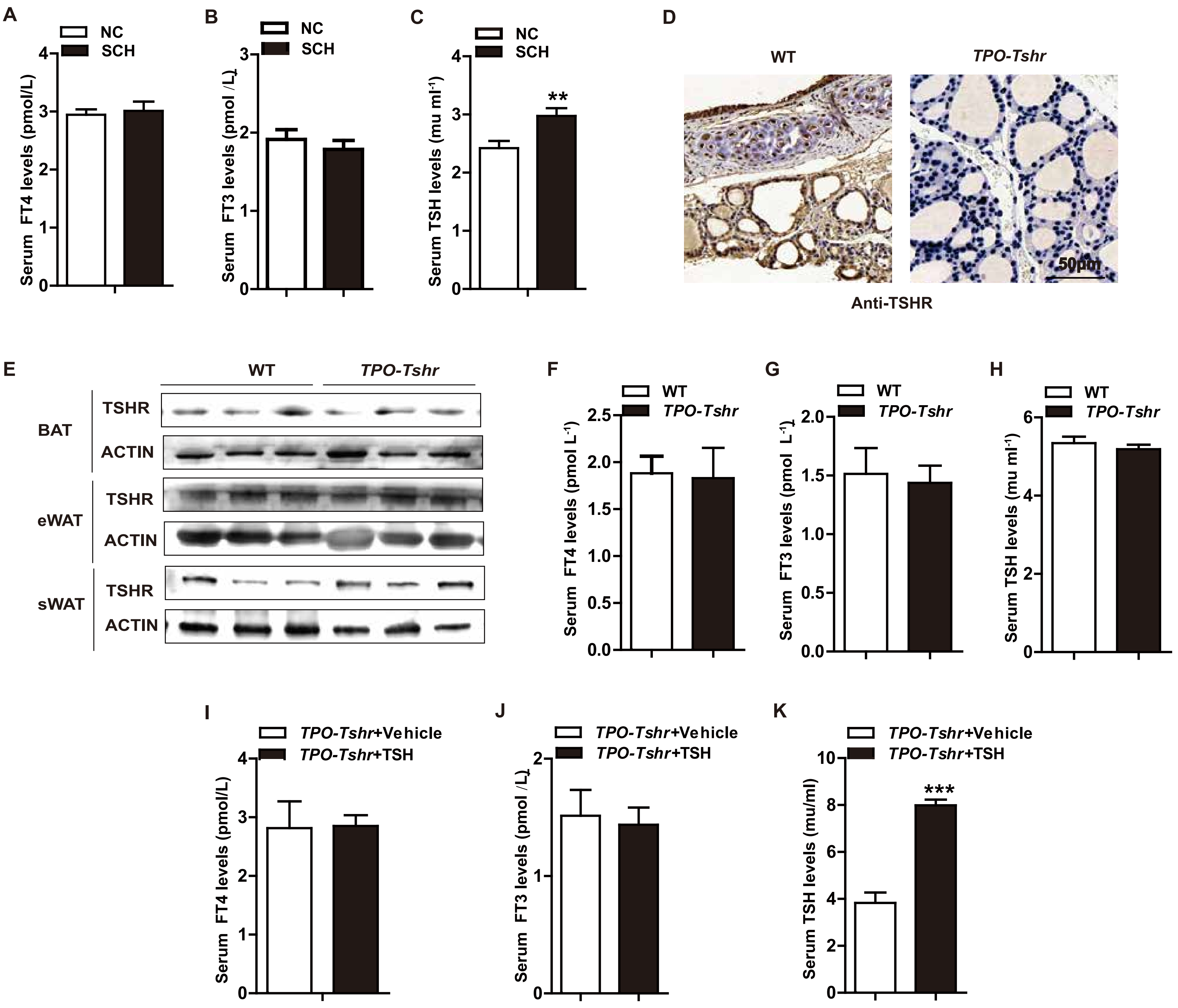

Supplementary Figure S2. The metabolic rate of TPO-Tshr mice is equivalent to that of their wild-type littermates

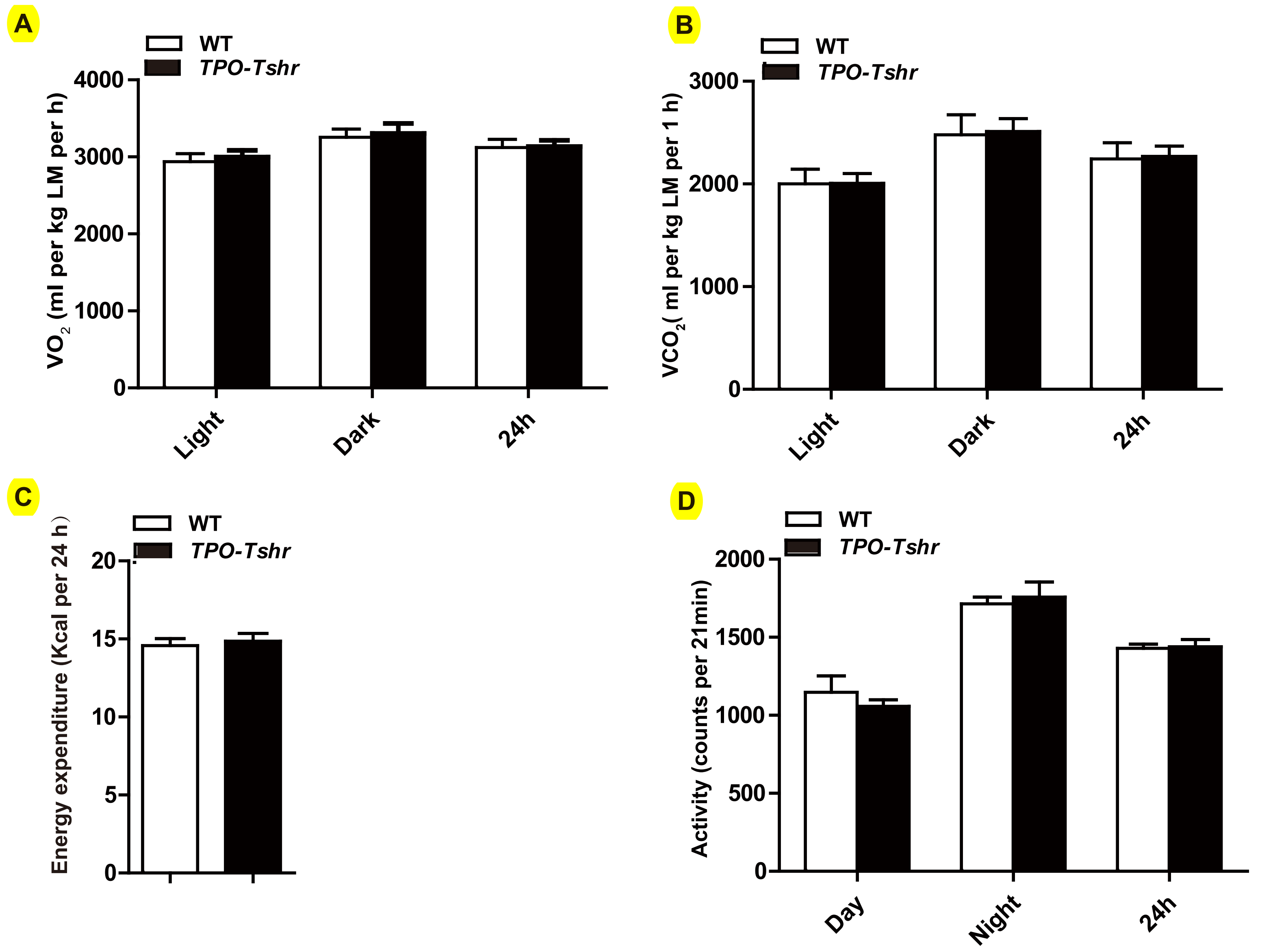

Supplementary Figure S4: Evaluation of the *FABP4-Tshr* and *Tshr*$^{-/-}$ mouse models

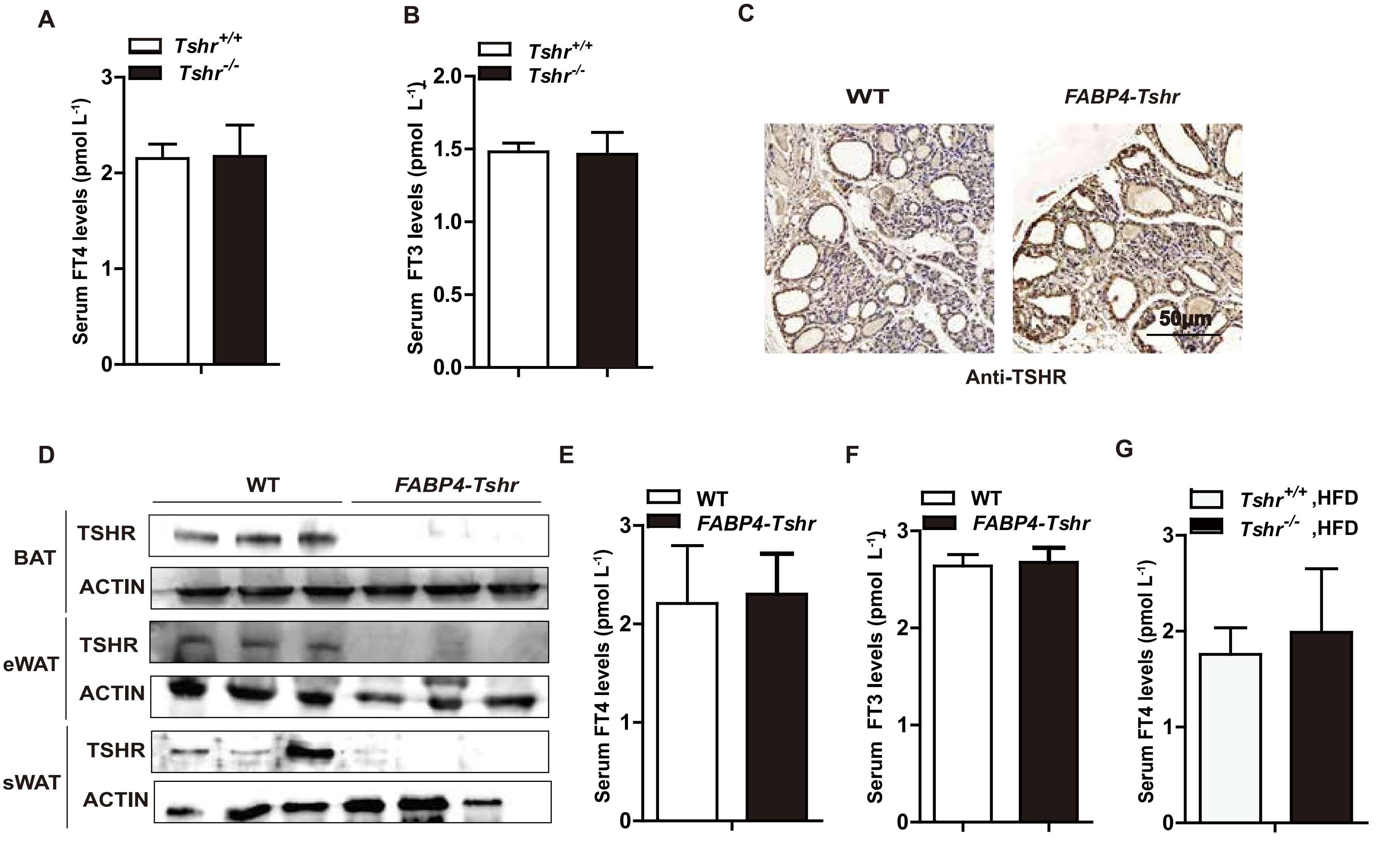

Supplementary Figure S4: *Tshr* knockout decreases BAT thermogenesis.

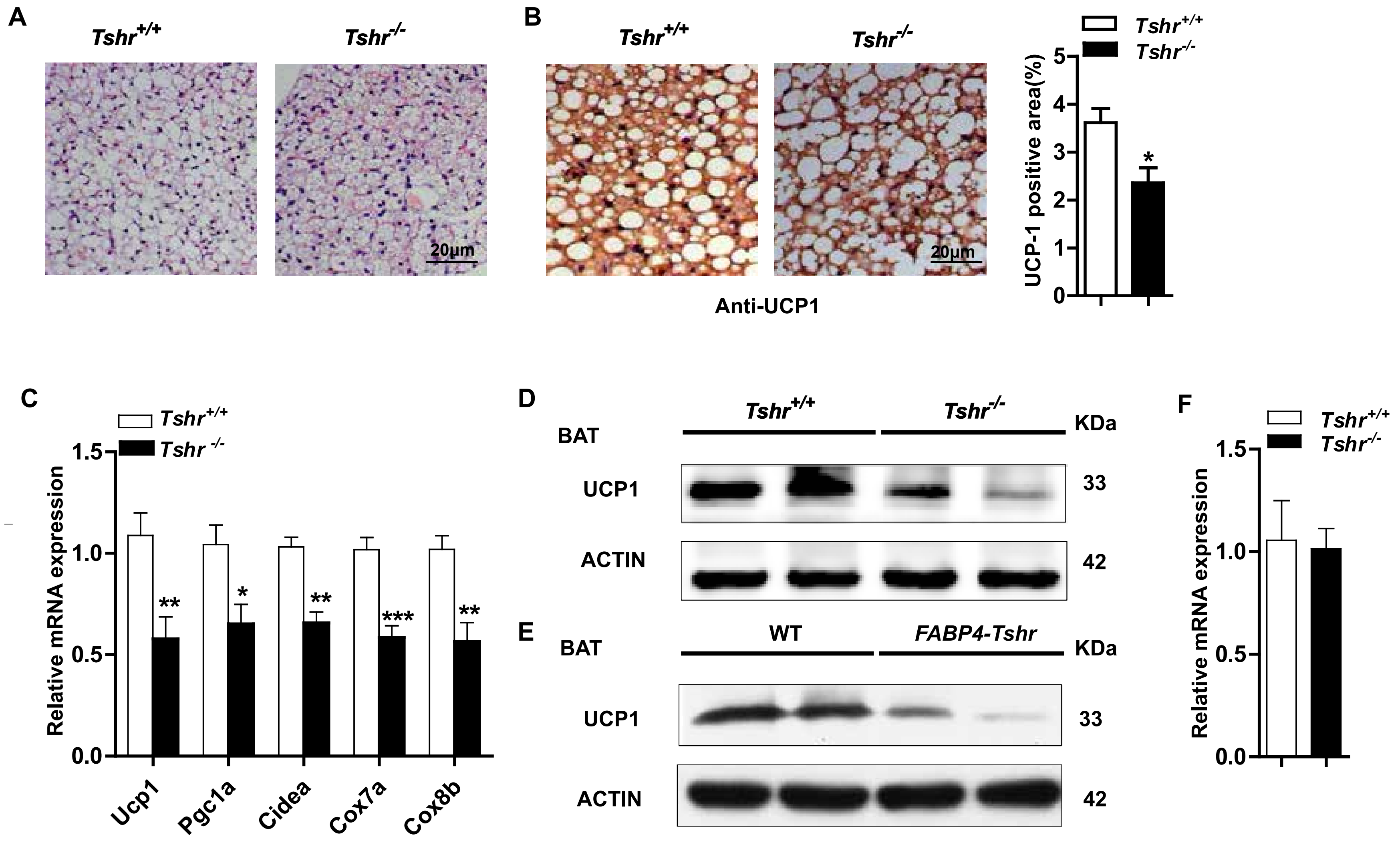

Supplement: Supplemental Material [file KADI_A_1783101_SM4510.zip › supplementary Figures.pdf]
