# Supplementary material for: TSH promotes adiposity by inhibiting the browning of white fat
Source: Adipocyte. 2020 Jun 24;9(1):264–78. doi: 10.1080/21623945.2020.1783101 (PMC7469524; doi:10.1080/21623945.2020.1783101)
Supplement: Supplemental Material [file KADI_A_1783101_SM4510.zip › Supplementary Table 1.docx]

**Supplementary Table 1. Primers used for real-time PCR analysis**

| **Gene name** | **Forward primer sequence (5'-3')** | **Reverse primer sequence (5'-3')** |
| --- | --- | --- |
| **mUCP1** | AGGCTTCCAGTACCATTAGGT | CTGAGTGAGGCAAAGCTGATTT |
| **mPGC1α** | AGCCGTGACCACTGACAACGAG | GCTGCATGGTTCTGAGTGCTAAG |
| **mCIDEA** | TGCTCTTCTGTATCGCCCAGT | GCCGTGTTAAGGAATCTGCTG |
| **mPRDM16** | CCACCAGCGAGGACTTCAC | GGAGGACTCTCGTAGCTCGAA |
| **mCOX7a1** | CAGCGTCATGGTCAGTCTGT | AGAAAACCGTGTGGCAGAGA |
| **mCOX8b** | TGTGGGGATCTCAGCCATAGT | AGTGGGCTAAGACCCATCCTG |
| **mFGF21** | AGGATGGAACAGTGGTAGGCG | GGCTTTGACACCCAGGATTTG |
| **mCEBPa** | TTGGTTTTGCTCTGATTCTTGC | CCACTTCATTTCATTGGTCCTCT |
| **mCEBPβ** | AGATGTTCCTGCGGGGTTG | CGAAACGGAAAAGGTTCTCAA |
| **mBMP4** | GGAGGAGGAAGAGCAGAGCC | TGAAGAGGAAACGAAAAGCAGA |
| **mBMP7** | GAAGTCCATCTCCGTAGTATCCG | CTCTGGTCACTGCTGCTGTTTT |
| **mPAT2** | TGCCACAAGAACGAGACGG | GAACACCAGAAGCCCCAGAAC |
| **mP2RX5** | GCATCATCCCCACAGTCATCA | TCTCAAACTTCTTGTCTCGGTAAAA |
