# Supplementary material for: TSH promotes adiposity by inhibiting the browning of white fat
Source: Adipocyte. 2020 Jun 24;9(1):264–78. doi: 10.1080/21623945.2020.1783101 (PMC7469524; doi:10.1080/21623945.2020.1783101)
Supplement: Supplemental Material [file KADI_A_1783101_SM4510.zip › Supplementary Table 2.docx]

**Supplementary Table 2. Antibody validation profile**

| **Primary Antibody** | **Clone** | **Company** | **Catalog No.** | **Dilution** |
| --- | --- | --- | --- | --- |
| UCP1 | Polyclonal | Proteintech | 23673-1-AP | 1:1000 (WB)  1:100 (IHC) |
| PGC1a | Polyclonal | Abcam | ab54481 | 1:1000 |
| β-actin | Polyclonal | Proteintech | 23660-1-AP | 1:7500 |
| CD137 | Polyclonal | Abcam | ab203391 | 1:100 |
| PRDM16 | Polyclonal | Abcam | ab106410 | 1:2500 |
| **Secondary antibody** | **Conjugate(s)**  **used** | **Company** | **Catalog No.** | **Dilution** |
| Anti-mouse IgG | HRP | Jackson | 111-035-003 | 1:200 |
| Anti-rabbit IgG | HRP | Jackson | 115-035-003 | 1:200 |
