# Supplementary material for: TSH promotes adiposity by inhibiting the browning of white fat
Source: Adipocyte. 2020 Jun 24;9(1):264–78. doi: 10.1080/21623945.2020.1783101 (PMC7469524; doi:10.1080/21623945.2020.1783101)
Supplement: Supplemental Material [file KADI_A_1783101_SM4510.zip › supplementary table3.docx]

Supplementary table 3:The primer sequences of the mouse genotyping

| genotype | primer sequences. |
| --- | --- |
| TSHR | Sequence 5' --> 3' Primer type |
|  | oIMR0872 - AAG TTC ATC TGC ACC ACC G - Mutant  oIMR1416 - TCC TTG AAG AAG ATG GTG CG - Mutant  oIMR3218 - CAG GGT GGA GAC GCA CAC TC - Wild type  oIMR3219 - AGA GAG TCC CAC AAC AGT C - Wild type |
| TPO-Cre | CRE-F: 5’-TGC CAC GAC CAA GTG ACA GCA ATG-3’  CRE-R: 5’-AGA GAC GGA AAT CCA TCG CTC G-3’ |
| Tshr-Loxp | SPH001_F1: 5’-GAGGATTTCTGTTGGTGGCTGG-3’  SPH001_R1: 5’-CACCCTTGATCCCCTTGACC-3’  SPH001_F2: 5’-GTAAACTGCTGGAGTACATGA-3’  SPH001_R2: 5’-AAAATTTAGCCTATGTGTAGCTT-3’ |
| FABP4-Cre | CRE-F: 5’-ATTTGCCTGCATTACCGGTC –3’  CRE-R: 5’-ATCAACGTTTTCTTTTCGG-3’ |
